# Associations of body mass index and waist circumference with risk of Guillain-Barré syndrome in women and men: A prospective analysis of three cohort studies

**DOI:** 10.1371/journal.pone.0239099

**Published:** 2020-12-01

**Authors:** Ming Ding, Andre Markon, Beverly Wolpert, Jorge E. Chavarro

**Affiliations:** 1 Department of Nutrition, Harvard School of Public Health, Boston, Massachusetts, United States of America; 2 Center for Food Safety and Applied Nutrition, Office of Analytics and Outreach, U.S. Food and Drug Administration, College Park, Maryland, United States of America; 3 Department of Epidemiology, Harvard School of Public Health, Boston, Massachusetts, United States of America; 4 Channing Division of Network Medicine, Brigham and Women’s Hospital and Harvard Medical School, Boston, Massachusetts, United States of America; McMaster University, CANADA

## Abstract

**Background:**

The association of body mass index (BMI) and waist circumference (WC) with risk of Guillain-Barré syndrome (GBS) has been inconsistent in previous studies.

**Methods:**

We examined the associations of BMI and WC in relation to risk of GBS among 252,980 participants from the Nurses’ Health Study (NHS), NHS-II, and the Health Professional Follow-up Study (HPFS). BMI and WC were assessed by self-reported questionnaire, and GBS cases were self-reported.

**Results:**

We documented 328 incident GBS cases during a total of 5,422,788 person years of follow-up. Compared to participants with BMI<25kg/m^2^, the multivariate pooled hazard ratio (HR) of GBS was 1.34 (95% CI: 1.04, 1.73) for overweight participants (25kg/m^2^≤BMI<30 kg/m^2^), and 1.68 (95% CI: 1.21, 2.35) for obese participants (BMI≥30 kg/m^2^) (P for trend = 0.001). Compared to participants with normal WC (<35 inches for women and <40 inches for men), the HR of GBS was 1.55 (95% CI: 1.10, 2.18) for participants with high WC (≥35 inches for women, and ≥40 inches for men). The positive associations of BMI and WC with risk of GBS were attenuated to null after mutually adjusting for BMI and WC. Joint analysis showed that the HR was 1.84 (95% CI: 1.27, 2.66) for participants with high WC and BMI≥25 kg/m^2^ in comparison to participants with normal WC and BMI<25kg/m^2^.

**Conclusion:**

These data from large cohorts showed that higher BMI and WC jointly were associated with higher risk of GBS. Our study highlighted the importance of maintaining a normal body weight and waist circumference in prevention of GBS.

## Introduction

The prevalence of obesity has increased dramatically in the past decades, and 1.9 billion adults worldwide were estimated to be overweight or obese in 2016 [1]. In the US alone, nearly 40% of adults have obesity [2]. In addition to its high frequency, obesity is a well characterized risk factor for multiple chronic diseases including cardiovascular disease [3], type 2 diabetes, and cancer [4], and is also an important risk factor for premature mortality [5]. As a result, obesity has become a leading public health concern worldwide. Although multiple biological mechanisms link obesity to its many adverse health outcomes, chronic low-grade systemic inflammation appears to be an important mechanism involved in chronic disease risk. Obesity has been associated with circulating inflammatory biomarkers, including higher concentrations of leptin, C-reactive protein (CRP), interleukin (IL)-6, and tumor necrosis factor-α (TNFα), and lower adiponectin [6]. In addition, increasing evidence suggests that obesity may also play a larger role in regulating immune response, as shown by multiple reports linking obesity to a higher risk of autoimmune diseases [7].

Guillain-Barré syndrome (GBS) is a rare an acute polyradiculoneuropathy of autoimmune origin. Clinically, GBS presents as rapidly evolving weakness and sensory disturbance in the arms, legs, facial, bulbar and respiratory muscles [8]. Most cases are preceded by an acute infectious disease [9], which may serve as a trigger for the manifestation of this disease. However, little is known regarding predisposing conditions that may increase the risk of developing GBS when exposed to known triggering events. Given the increasing evidence showing the role of obesity as a risk factor for autoimmune and immune-mediated conditions [7], we examined the association of body mass index (BMI) and waist circumference (WC) with risk of GBS in three large prospective cohorts of women and men living in North America. We hypothesized that obesity and abdominal obesity would be risk factors for developing GBS.

## Methods

### Study population

The Nurses’ Health Study (NHS) began in 1976, when 121,700 female registered nurses aged 30–55 y residing in 11 states were recruited to complete a baseline questionnaire about their lifestyle and medical history. The NHS II was established in 1989 and consisted of 116,671 female registered nurses aged 25–42 y at baseline. The Health Professional Follow-up Study (HPFS) was initiated in 1986, and was composed of 51,529 male dentists, pharmacists, veterinarians, optometrists, osteopathic physicians, and podiatrists, aged 40–75 y at baseline. In the three cohorts, questionnaires were collected at baseline and biennially thereafter, to update information on lifestyle factors and the occurrence of chronic diseases. Approximately 95% of the participants in the three cohorts identify as Caucasians. For the current analysis, we excluded participants who reported a history of GBS at baseline (1982 for the NHS, 1989 for the NHS-II, and 1986 for the HPFS). Return of a completed questionnaire was considered as implied consent to participate. The study protocol was approved by the institutional review boards of the Brigham and Women’s Hospital and Harvard T.H. Chan School of Public Health, and those of participating registries as required. The study has been performed in accordance with the ethical standards laid down in the 1964 Declaration of Helsinki and its later amendments.

### Assessment of body mass index and waist circumference

In the three cohorts, height and weight were self-reported at baseline, and body weight was asked by questionnaire every two years. BMI was calculated as weight (kg) over height (m) squared. Women were asked to report their waist and hip circumferences using a tape measure included in the questionnaire in 1986, 1996 and 2000 in the NHS, and 1993 in the NHS-II, and 1987, 1996, 2008 in the HPFS. Self-reported weight was previously validated in a subgroup of 140 NHS participants and 123 HPFS participants. The correlation between measured and self-reported weight was 0.97 for both NHS and HPFS participants, and the correlation between measured and self-reported waist circumference was 0.89 and 0.95 in women and men, respectively [10].

### Assessment of covariates

In the biennial follow-up questionnaires, updated information was collected on age, weight, smoking status, physical activity, medication use, and self-reported diagnosis of hypertension and hypercholesterolemia until 2010. For NHS and NHS-II participants, we also ascertained data on menopausal status and postmenopausal hormone use. Dietary intakes were assessed by food frequency questionnaire (FFQ), which has shown good validity and reproducibility [11–14]. We derived the Alternate Healthy Eating Index 2010 (AHEI-2010) as a summary measure of diet quality [15], whose total score ranged from 0 to 110 with a higher score indicating better diet quality.

### Assessment of GBS cases

At baseline and in each follow-up questionnaire, participants were asked to report any disease newly diagnosed by a physician by choosing from a list of common diseases or by writing in their diagnosis if it was not part of the disease list. GBS was never offered as an option in the disease list and therefore all reports are of participants who wrote-in their diagnosis. All write-in diagnoses were reviewed and assigned an ICD code. We identified GBS cases with ICD-8 code 354.0.

### Statistical analysis

We calculated each individual’s person-time from the date of the return of the baseline questionnaire to the date of death or the end of follow-up (31 December 2014 for the NHS, 2015 for the NHS-II, and 2016 for the HPFS), whichever came first. We used Cox proportional hazards regression models to examine the associations of BMI and WC with risk of GBS. The regression models included calendar time in 2-y intervals as the time scale, and were stratified by age in years. We used baseline BMI and WC measured at the earliest time as main exposure. In the multivariable analysis, we further adjusted for smoking status, physical activity, AHEI, alcohol intake, and total energy intake. We additionally adjusted for menopausal status and postmenopausal hormone use among women. To examine associations of BMI and WC with risk of GBS, we included BMI and WC simultaneously in the model in main analysis. We used residual method to test the robustness of our findings in sensitivity analysis, which is a method commonly used in nutrition epidemiology [16]. In this method, we regressed WC on BMI to obtain residuals of WC, which is calculated as differences between each individual's WC and the WC predicted by BMI. Thus, the residual of WC is independent of BMI, and the correlation between WC and BMI was accounted for. We examined associations of BMI and residual of WC on risk of GBS. All analyses were performed separately in each cohort, and then pooled to obtain the overall hazard ratio using a fixed-effects model. We used random-effects models if significant heterogeneity across cohorts was found. Statistical heterogeneity across studies was assessed by Cochrane Q test, with P <0.1 indicating significant between-study heterogeneity [17]. All statistical tests were 2-sided and performed using SAS version 9.2 for UNIX (SAS Institute Inc).

## Results

The analysis included 252,980 participants, of whom 88,640 participants were from the NHS, 114,588 participants from the NHS-II, and 49,752 participants from the HPFS. **Table 1** shows the baseline characteristics of the participants by BMI categories. Those who had BMI <25 kg/m^2^ were more likely to be physically active, were less likely to be current smokers, and had a healthier diet.

**Table 1:** Age-adjusted characteristics of participants in the NHS, NHS-II, and HPFS according to BMI categories.

	NHS (1984)			NHS2 (1989)			HPFS (1986)		
BMI (kg/m^2^)	≤24.9	25–29.9	≥ 30	≤24.9	25–29.9	≥ 30	≤24.9	25–29.9	≥ 30
Number of participants	52499	23957	12184	80059	21364	13165	23057	22590	4105
Age (year)	48.0 (7.3)	49.5 (7.1)	49.0 (7.0)	35.8 (4.7)	36.7 (4.6)	37.2 (4.5)	54.0 (10.2)	54.7 (9.6)	54.2 (9.1)
Physical activity (MET-h/wk)	15.7 (22.4)	12.5 (18.8)	10.1 (16.3)	26.9 (38.8)	21.6 (31.8)	17.9 (28.9)	24.0 (31.6)	19.1 (26.8)	13.0 (21.9)
Total energy intake (kcal/d)	1735 (524)	1737 (531)	1790 (558)	1774 (540)	1807 (557)	1852 (576)	1991 (606)	1977 (626)	2020 (657)
AHEI	48.3 (11.1)	47.6 (10.5)	45.7 (10.2)	49.1 (11.1)	48.0 (10.7)	46.0 (10.6)	53.9 (11.8)	52.1 (11.2)	50.6 (11.2)
Alcohol (g/d)	7.9 (11.8)	6.0 (10.6)	4.1 (9.5)	3.5 (6.3)	2.6 (5.8)	1.8 (4.9)	11.3 (14.9)	11.6 (15.8)	10.2 (16.0)
Never smokers, %	58	55	52	35	36	34	52	58	60
Postmenopausal women, %	43	42	42	3	3	4	NA	NA	NA
Postmenopausal hormone use, (% among total women)	11	9	6	3	3	3	NA	NA	NA

BMI: body mass index; AHEI: alternative healthy eating index.

In total, 328 GBS cases were documented during 5,422,788 person years of follow-up in the three cohorts. As expected, the incident rate of GBS was higher in men than women. Compared to participants with BMI <25kg/m^2^, the multivariate pooled hazard ratio (HR) of GBS was 1.34 (95% CI: 1.04, 1.73) for participants with overweight (25kg/m^2^≤BMI<30 kg/m^2^), and 1.68 (95% CI: 1.21, 2.35) for participants with obesity (BMI≥30 kg/m^2^) (P for trend = 0.001) (**Table 2**). Compared to participants with normal WC (<35 inches for women and <40 inches for men), the HR of GBS was 1.55 (95% CI: 1.10, 2.18) for participants with high WC (≥35 inches for women, and ≥40 inches for men) (**Table 3**). The associations of BMI and WC with risk of GBS did not differ across cohorts (P for heterogeneity >0.1). The positive associations of BMI and WC with risk of GBS were attenuated after mutually adjusting for each other. Obesity remained associated with a significantly higher risk of GBS using the residual method. However, WC was no longer associated with risk of GBS (P for trend = 0.30), and the HR of GBS was 1.03 (95% CI: 0.98, 1.09) per inch increase in WC using residual method.

**Table 2:** Associations of baseline BMI and risk of incident GBS in the three cohorts.

	BMI ≤24.9	BMI 25–29.9	BMI ≥ 30	P for trend
*NHS*				
Number of cases/person year	54/1447788	40/643225	19/314137	
Age-adjusted	1.00	1.68 (1.11, 2.53)	1.57 (0.93, 2.65)	0.02
Multivariate-adjusted	1.00	1.58 (1.03, 2.43)	1.45 (0.83, 2.53)	0.07
Additionally adjusting for WC	1.00	0.72 (0.33, 1.56)	1.02 (0.37, 2.83)	0.85
Additionally adjusting for WC using residual method	1.00	0.75 (0.37, 1.55)	1.61 (0.74, 3.51)	0.50
*NHS2*				
Number of cases/person year	55/1441989	20/379123	19/228916	
Age-adjusted	1.00	1.37 (0.82, 2.29)	2.15 (1.27, 3.64)	0.004
Multivariate-adjusted	1.00	1.40 (0.82, 2.38)	2.28 (1.32, 3.96)	0.004
Additionally adjusting for WC	1.00	2.94 (1.33, 6.48)	1.47 (0.36, 6.00)	0.14
Additionally adjusting for WC using residual method	1.00	3.02 (1.48, 6.18)	1.39 (0.40, 4.85)	0.06
*HPFS*				
Number of cases/person year	52/455164	62/437304	14/75142	
Age-adjusted	1.00	1.21 (0.83, 1.74)	1.69 (0.93, 3.05)	0.09
Multivariate-adjusted	1.00	1.21 (0.83, 1.76)	1.57 (0.86, 2.87)	0.12
Additionally adjusting for WC	1.00	1.15 (0.69, 1.91)	1.93 (0.80, 4.65)	0.22
Additionally adjusting for WC using residual method	1.00	1.19 (0.75, 1.90)	2.10 (1.05, 4.19)	0.07
*Pooled*				
Age-adjusted	1.00	1.37 (1.07, 1.75)	1.74 (1.26, 2.39)	<0.001
Multivariate-adjusted	1.00	1.34 (1.04, 1.73)	1.68 (1.21, 2.35)	0.001
Additionally adjusting for WC	1.00	1.25 (0.86, 1.82)	1.50 (0.82, 2.74)	0.14
Additionally adjusting for WC using residual method	1.00	1.29 (0.91, 1.82)	1.78 (1.10, 2.87)	0.02

Multivariate model adjusted for age, smoking status, physical activity, diet quality, alcohol intake, total energy intake, menopausal status, and postmenopausal hormone use.

**Table 3:** Associations of baseline waist circumference with hazard ratio of GBS in the three cohorts.

	WC<35 inches for women, WC<40 inches for men	WC≥35 inches for women, WC≥40 inches for men	P for trend
*NHS*			
Number of cases/person year	41/1212177	15/253056	
Age-adjusted	1.00	1.79 (0.99, 3.25)	0.05
Multivariate-adjusted	1.00	1.82 (0.98, 3.38)	0.06
Additionally adjusting for BMI	1.00	2.02 (0.87, 4.68)	0.10
*NHS2*			
Number of cases/person year	30/784717	9/180980	
Age-adjusted	1.00	1.36 (0.64, 2.88)	0.42
Multivariate-adjusted	1.00	1.67 (0.77, 3.63)	0.20
Additionally adjusting for BMI	1.00	1.08 (0.43, 2.72)	0.89
*HPFS*			
Number of cases/person year	59/518045	25/146386	
Age-adjusted	1.00	1.48 (0.92, 2.37)	0.10
Multivariate-adjusted	1.00	1.43 (0.88, 2.31)	0.15
Additionally adjusting for BMI	1.00	1.11 (0.60, 2.06)	0.73
*Pooled*			
Age-adjusted	1.00	1.52 (1.09, 2.12)	0.02
Multivariate-adjusted	1.00	1.55 (1.10, 2.18)	0.01
Additionally adjusting for BMI	1.00	1.27 (0.81, 1.97)	0.30

Multivariate model adjusted for age, smoking status, physical activity, diet quality, alcohol intake, total energy intake, menopausal status, and postmenopausal hormone use.

**Fig 1:**
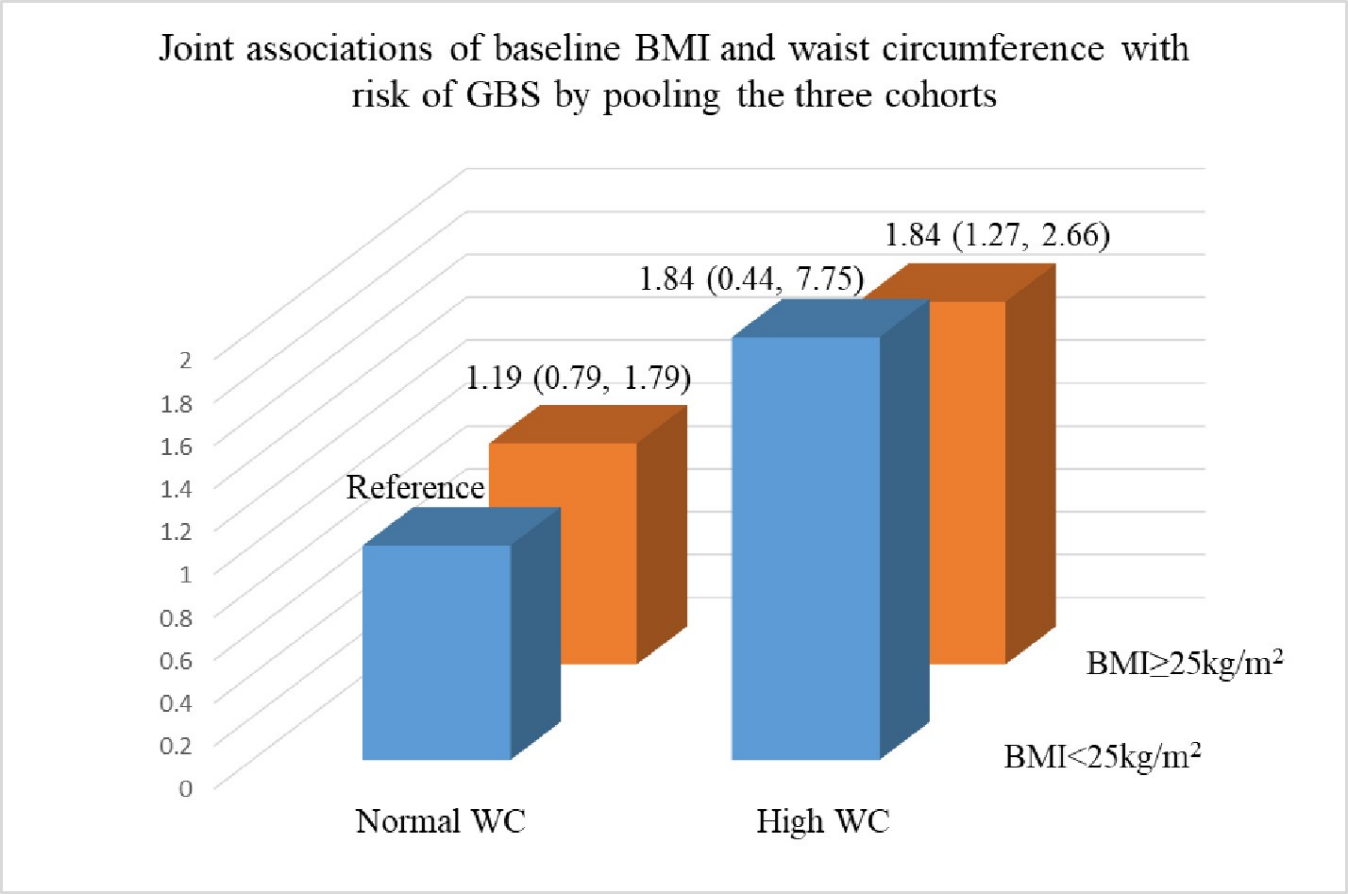
Joint associations of baseline BMI and waist circumference with risk of GBS by pooling the three cohorts. Multivariate model adjusted for age, smoking status, physical activity, diet quality, alcohol intake, total energy intake, menopausal status, and postmenopausal hormone use.

Analyses jointly classifying participants according to their BMI and WC suggested that WC may be a more important risk factor for GBS than BMI (**Fig 1**). Individuals with high WC and overweight or obesity had a HR for GBS of 1.84 (95% CI: 1.27, 2.66) relative to participants with normal WC and BMI<25 kg/m^2^; the estimate for individuals with BMI<25 kg/m^2^ and high WC was similar but it was based on very sparse data (n = 2 cases).

## Discussion

In the present analysis, which included three large ongoing cohort studies involving 252,980 participants followed for 27–34 years and 328 incident GBS cases showed that higher BMI and WC were associated with higher risk of GBS. To our knowledge, this is the first time that obesity has been linked to an excess risk of GBS and therefore our findings should be interpreted with caution. Moreover, a reexamination of the reported associations in independent cohorts is necessary. Nevertheless, should our findings be replicated, they open the door to better understanding how nutritional status may affect the baseline susceptibility to develop GBS after a trigger event.

The literature of the relation between adiposity and risk of GBS is scant. Only one prospective cohort study has previously evaluated the relation between anthropometric measures of adiposity and risk of GBS. In the Danish National Birth Cohort, which followed 75,008 women for a median 11 years, BMI was not associated with risk of GBS [18]. However, only 14 GBS cases were documented in this study, resulting in very limited statistical power. Moreover, data on WC, which our data suggests may be as or more important than BMI, was lacking. While we are unaware of other studies evaluating the relation between adiposity and GBS in general-population cohorts, GBS has been documented as a rare complication of bariatric surgery [19, 20], although it is unclear if these cases are due to the indication of surgery (severe obesity), to nutritional deficiencies secondary to the bariatric procedure [19], or to undergoing surgery itself [21]. Clearly, additional research on the relation between obesity and GBS is needed.

An association between excess adiposity and GBS is biologically plausible. Prospective cohort studies have shown that higher BMI was associated with higher plasma concentrations of CRP, IL-6, TNF-α, and leptin and lower adiponectin [6]. In the process of inflammation, visceral adipocytes have played an important role. When excessive lipid stores in the body, visceral adipocytes secrete increasing amounts of inflammatory cytokines including CRP, IL-6 and TNF-α. These inflammatory factors attract macrophages to migrate to the adipose tissue, promoting the release of cytokine and obesity-associated inflammation including leptin and adiponectin [22]. In the early stages of GBS, the cytokines and adipokines released by macrophages contributed to the enhancement of blood nerve barrier permeability and the migration of T cells into the peripheral nervous system, resulting in demyelination of peripheral nerves and autoantibodies against gangliosides [23].

Another explanation for the positive association between BMI and risk of GBS might be due to shared mechanisms of GBS with other autoimmune diseases. It has been previously documented that experiencing immune-mediated diseases including multiple sclerosis, type 1 diabetes and rheumatological disorders early in life is associated with significantly higher risk of GBS [24]. In addition, there is growing evidence for a causal role of obesity in the etiology of autoimmune and immune-mediated diseases. For example, observational studies have shown that obesity is associated with higher risk of multiple sclerosis [25], and Mendelian randomization have demonstrated that genetically elevated BMI is in turn associated with risk of multiple sclerosis [26, 27]. Obesity has been also been related to higher risk of Crohn’s disease [28] and Type 1 diabetes mellitus [29]. Moreover, both obesity and abdominal obesity have been independently associated with risk of rheumatoid arthritis [30, 31]. Furthermore, pre-diagnostic levels of plasma CRP and IL-6 are associated with a higher risk of Crohn’s disease [32]; and TNFR2 level, a proxy for TNF-α, is associated with higher risk of rheumatoid arthritis up to 12 years prior to disease symptoms [33]. Therefore, it was possible that the positive associations of BMI and WC with risk of GBS are a reflection of a broader role of obesity in regulating immune function and elevating the risk of autoimmune diseases generally.

A third possible explanation for the observed positive association between obesity and risk of GBS is that obesity is a risk factor for infections known to be antecedents of GBS symptoms. *C jejuni* is the predominant infection associated with GBS symptom onset accounting for 25–50% of the adult GBS cases [8]. *C jejuni* has lipooligosaccharides in the bacterial wall that mimic gangliosides, resulting in activated T cells attacking both the bacteria and peripheral nerves [34]. However, fewer than one per 1,000 patients with *C*. *jejuni* infection develop GBS, indicating that patients’ factors play a significant role in the pathologic process [8]. Moreover, while previous work has shown that people with obesity may experience an elevated risk of nosocomial and surgical site infections [35], and a previous report from these cohorts showed an elevated risk of community acquired pneumonia among individuals with obesity [36]. It is not clear to what extent obesity may or may not be a risk factor for other common community acquired infectious diseases [35], including infections associated with GBS.

It is also important to consider the possibility that the observed relations are spurious. An argument against interpreting these findings as reflective of a true biological relation between adiposity and GBS is the fact that, to our knowledge, the incidence rate of GBS has not increased as the prevalence of obesity has increased across the globe. If there were a causal link between obesity and GBS, an increase in the frequency of GBS in the US would have been expected to take place between the mid-1970s and the late 1990s, when the prevalence of obesity increased at the highest rate since population prevalence data is available [37]. However, a change in the incidence of GBS would be very difficult to detect, given the low incidence of GBS, the modest size of the association between obesity and GBS suggested by this study, the changes in the age distribution of the US population, and changes in standards for documenting GBS in population statistics during this period. Thus, the possibility that our results are a chance finding should be equally considered until confirmed or refuted by independent studies.

Our study has several notable strengths. First, our study is a well-characterized large cohort of women followed prospectively for more than 30 years. As GBS is a disease with low incidence rate, our large sample size and extended follow-up improved power to detect meaningful associations. Second, in our analysis, we collected data on BMI and other anthropometrics such as waist measurement which allowed us to evaluate the associations of overall obesity and abdominal obesity with risk of GBS. Furthermore, BMI and WC was collected prior to diagnosis of GBS, hence avoiding reverse causation and recall bias. Moreover, validation studies have demonstrated that self-reported weight and waist circumferences from our participants are highly valid and reproducible [10]. Third, we collected detailed and validated information on potentially important confounders such as dietary intakes, physical activity, smoking status, postmenopausal status, and hormone use, which allowed us to appropriately control for a variety of risk factors that may have influenced our observed associations.

Several limitations of our study also need to be considered. First, the GBS cases were self-reported, opening the door to misclassification of GBS diagnoses. However, as assessment of GBS was independent of the assessment of anthropometric characteristics and anthropometric assessment predated outcome assessment, we would expect a non-differential misclassification of outcomes in relation to exposure, which could result in a bias towards the null and less precise estimates. Second, the NHS, NHS-II, and HPFS are predominantly white health professionals which may limit the generalizability of the findings. However, their occupational status is a distinct advantage that allows us to collect high-quality data using self-reported questionnaires and enhance the internal validity of the study by reducing confounding. Last, as is the case in all observational studies, despite our ability to adjust for a variety of potential confounders, we cannot rule out the possibility of residual confounding.

In conclusion, we found that higher BMI and WC were associated with higher risk of GBS independently of major lifestyle factors. Further, stratified analyses suggested that waist circumference may be a more important risk factor for GBS than overall obesity. If replicated in independent studies, these findings could open the door to better understanding how nutritional status may impact the baseline susceptibility to GBS. Nevertheless, given the novelty of these findings and the limitations of our study, it is exceedingly important that this relation is independently evaluated to confirm or refute our findings.

## Supporting information

S1 Table Joint associations of baseline BMI and waist circumference with risk of GBS in the three cohorts. Multivariate model adjusted for age, smoking status, physical activity, diet quality, alcohol intake, total energy intake, menopausal status, and postmenopausal hormone use.
(DOCX)
