# Supplementary material for: Associations of body mass index and waist circumference with risk of Guillain-Barré syndrome in women and men: A prospective analysis of three cohort studies
Source: PLoS One. 2020 Dec 1;15(12):e0239099. doi: 10.1371/journal.pone.0239099 (PMC7707592; doi:10.1371/journal.pone.0239099)
Supplement: S1 Table — Multivariate model adjusted for age, smoking status, physical activity, diet quality, alcohol intake, total energy intake, menopausal status, and postmenopausal hormone use. (DOCX) [file pone.0239099.s001.docx]

**S1 Table. Joint associations of baseline BMI and waist circumference with risk of GBS in the three cohorts.**

|  | BMI<25kg/m^2^,  WC<35 inches for women, WC<40 inches for men | BMI<25kg/m^2^,  WC≥35 inches for women, WC≥40 inches for men | BMI≥25kg/m^2^,  WC<35 inches for women, WC<40 inches for men | BMI≥25kg/m^2^,  WC≥35 inches for women, WC≥40 inches for men |
| --- | --- | --- | --- | --- |
| *NHS* |  |  |  |  |
| Number of cases/person year | 35/869765 | 0/24913 | 5/245601 | 15/208036 |
| Age-adjusted | 1.00 | NA | 0.53 (0.21, 1.37) | 1.90 (1.03, 3.49) |
| Multivariate-adjusted | 1.00 | NA | 0.54 (0.21, 1.38) | 1.97 (1.04, 3.74) |
| *NHS2* |  |  |  |  |
| Number of cases/person year | 24/682922 | 0/44170 | 6/98131 | 9/135578 |
| Age-adjusted | 1.00 | NA | 2.10 (0.85, 5.18) | 2.07 (0.91, 4.66) |
| Multivariate-adjusted | 1.00 | NA | 2.26 (0.91, 5.61) | 2.49 (1.07, 5.78) |
| *HPFS* |  |  |  |  |
| Number of cases | 34/313951 | 2/9922 | 25/195108 | 23/133168 |
| Age-adjusted | 1.00 | 1.83 (0.44, 7.67) | 1.20 (0.71, 2.01) | 1.58 (0.93, 2.69) |
| Multivariate-adjusted | 1.00 | 1.84 (0.44, 7.75) | 1.23 (0.73, 2.07) | 1.54 (0.89, 2.65) |

Multivariate model adjusted for age, smoking status, physical activity, diet quality, alcohol intake, total energy intake, menopausal status, and postmenopausal hormone use.
